# Attitudes of Jordanian Anesthesiologists and Anesthesia Residents towards Artificial Intelligence: A Cross-Sectional Study

**DOI:** 10.3390/jpm14050447

**Published:** 2024-04-25

**Authors:** Isam Bsisu, Rami Alqassieh, Abdelkarim Aloweidi, Abdulrahman Abu-Humdan, Aseel Subuh, Deema Masarweh

**Affiliations:** 1 Department of Anesthesia and Intensive Care, School of Medicine, The University of Jordan, Amman 11942, Jordan; akaloweidi@hotmail.com (A.A.); abdo3azzam@gmail.com (A.A.-H.); deema.masarweh@gmail.com (D.M.); 2 UCSF Center for Health Equity in Surgery and Anesthesia, San Francisco, CA 94158, USA; 3 Department of Anesthesia and Intensive Care, Arab Medical Center, Amman 11181, Jordan; 4 Department of General Surgery and Anesthesia and Urology, Faculty of Medicine, The Hashemite University, Zarqa 13133, Jordan; rami_qaisieh@yahoo.com; 5 Department of Internal Medicine, School of Medicine, The University of Jordan, Amman 11942, Jordan; aseelsobeh@yahoo.com

**Keywords:** anesthesia, perioperative medicine, artificial intelligence

## Abstract

Success in integrating artificial intelligence (AI) in anesthesia depends on collaboration with anesthesiologists, respecting their expertise, and understanding their opinions. The aim of this study was to illustrate the confidence in AI integration in perioperative anesthetic care among Jordanian anesthesiologists and anesthesia residents working at tertiary teaching hospitals. This cross-sectional study was conducted via self-administered online questionnaire and includes 118 responses from 44 anesthesiologists and 74 anesthesia residents. We used a five-point Likert scale to investigate the confidence in AI’s role in different aspects of the perioperative period. A significant difference was found between anesthesiologists and anesthesia residents in confidence in the role of AI in operating room logistics and management, with an average score of 3.6 ± 1.3 among residents compared to 2.9 ± 1.4 among specialists (*p* = 0.012). The role of AI in event prediction under anesthesia scored 3.5 ± 1.4 among residents compared to 2.9 ± 1.4 among specialists (*p* = 0.032) and the role of AI in decision-making in anesthetic complications 3.3 ± 1.4 among residents and 2.8 ± 1.4 among specialists (*p* = 0.034). Also, 65 (55.1%) were concerned that the integration of AI will lead to less human–human interaction, while 81 (68.6%) believed that AI-based technology will lead to more adherence to guidelines. In conclusion, AI has the potential to be a revolutionary tool in anesthesia, and hesitancy towards increased dependency on this technology is decreasing with newer generations of practitioners.

## 1. Introduction

In this continuously evolving scientific world, there is constant effort to integrate advanced technologies into our daily lives to improve the quality of our lives and the services we provide [1]. Notably, the medical field has witnessed substantial development, with anesthesiology being an example of heightened safety and the facilitation of more complex surgeries due to the advancements in pharmacology, medical engineering, and computer science [2]. Now we find ourselves on the precipice of a new advancement that could revolutionize the field, the introduction of artificial intelligence (AI) [3], which is defined as a sophisticated set of algorithms constructed to mimic human cognitive processes, giving them the ability to reason and perform functions such as object recognition, problem-solving, and evidence-based decision-making capabilities [3,4]. Another important feature of AI is the ability to learn; that is, modification of actions based on previous experiences and acquired knowledge [4].

The use of AI in anesthesia has been around for years. In the 1990s, anesthesiologists used AI to aid in the safe infusion of propofol while maintaining adequate depth of anesthesia during surgery. The Diprifusor system was designed with the pharmacological properties of propofol in mind to maintain adequate plasma levels of the drug [5]. Another example is closed-loop anesthesia drug administration integrating the effects of combining remifentanil and propofol into its protocol to help provide safe drug infusion rates to maintain surgical anesthetic depth [6]. Alas, the number and variety of AI-assisted technologies has in reality became more widely used only recently [7].

In the last few years, AI has been used by anesthesiologists to help with intraoperative monitoring, anesthesia risk predictions, and analysis of data [8]. Anesthesia as a specialty is uniquely positioned to benefit from AI in many ways. AI can be integrated in pain management protocols, drug delivery systems, and in critical care monitoring [9]. In addition, there is emerging literature that encourages the application of AI among special patients’ groups, such as in pediatric anesthesia, which holds potential for ultimately improving patient safety and outcomes [10].

A hinderance to the spread and incorporation of AI technology is understanding and proper utilization on the part of the user. Although AI has found itself becoming increasingly used in anesthesia, little is known about the attitudes of medical professionals towards the integration of AI into their daily practice [11,12]. This paper aims to illustrate the confidence in AI integration in different aspects of perioperative anesthetic care and the general concerns about AI integration among Jordanian anesthesiologists and anesthesia residents working at the tertiary teaching hospitals of the Ministry of Health in Jordan.

## 2. Materials and Methods

### 2.1. Study Design

This cross-sectional study was conducted between 20 October 2023 and 30 November 2023 using an online structured self-administered questionnaire. The targeted population was Jordanian anesthesiologists and anesthesia residents working at tertiary referral teaching hospitals of the Ministry of Health in Jordan. We excluded anesthesia technicians, nurse anesthetists, and anesthesiologists working in non-teaching hospitals, as well as those working in the private sector and military hospitals.

### 2.2. Questionnaire

We designed an online structured self-administered questionnaire using Google Forms, which is an online web-based survey creator software developed by Google. The questionnaire was divided into two sections. The first section investigated demographics and experience in the field of anesthesia. The second section investigated attitudes toward artificial intelligence in different aspects in the field of anesthesia. We used a five-point Likert scale to investigate the confidence in AI role in different aspects of the perioperative period, where 5 meant “very useful”, while 1 meant “not useful at all”.

The Ministry of Health was contacted, and they stated that there are currently 104 board-certified anesthesiologists working as anesthesia and intensive care specialists in their hospitals. There were 169 anesthesia resident physicians. In addition, there were 165 board-eligible anesthesia physicians and 396 anesthesia technicians, which were excluded from this study. The participants were reached via their institutional emails and phone numbers, with a reminder being sent two weeks after they were first contacted. We received an overall of 118 responses from 44 anesthesiologists and 74 anesthesia residents. The response rate was 42.3% for specialists and 43.8% for resident physicians.

A pilot test of the survey was conducted first by filling the questionnaire by 3 anesthesiologists and 4 anesthesia residents and modifying the questionnaire based on their feedback. Reliability statistical calculation was conducted on the 118 responses received, including all aforementioned Likert scales, and Cronbach’s alpha was 0.943, which indicates a high level of internal consistency for our scale with this specific sample. Moreover, none of the questions would substantially affect reliability or result in a higher Cronbach’s alpha if they were deleted (Table S1).

### 2.3. Ethical Approval

The study was approved by the Institutional Review Board (IRB) committee of Hashemite University (approval 1/2/2023/2024). Informed consent was obtained in the introduction page of the online questionnaire by checking (ticking) the approval box, after which respondents were able to proceed to the questionnaire. We did not include personal information in the questionnaire form, and the collected data were used exclusively for statistical analysis.

### 2.4. Statistical Analysis

We used Statistical Package for the Social Sciences (SPSS) version 25.0 for statistical analysis. Descriptive statistics were applied, and data are presented as means ± standard deviation for numeric variables, and number (percentage) for categorical variables. The Mann–Whitney U test was used for the comparison between anesthesia specialists and residents for numeric variables, including the five-point Likert scale. The chi-squared test was used to compare between the aforementioned two groups in terms of categorical variables. A two-sided *p*-value < 0.05 was used as the significance threshold in all abovementioned statistical tests.

## 3. Results

Overall, 118 responses were collected from 44 anesthesiologists and 74 anesthesia residents (Table 1). The mean age of the included resident physicians was 28.6 ± 3.0 years, while the mean age of the included specialists was 39.7 ± 9.5 years (*p* < 0.001), with an average experience of 7.7 ± 8.8 years as a board anesthesiologist. Of the studied sample, 69 (58.5%) had previous computer programming experience. Only 45 (38.1%) had previously read a scientific article about AI prior to enrollment in this study, and 74 (62.7%) of them had not previously used either AI-based software or AI-based technology.

Using a five-point Likert scale, we compared the resident physicians and specialists in terms of their confidence in AI role in different perioperative roles, as illustrated in Table 2. We found a significant difference in the confidence in the role of AI in operating room logistics and management, with an average score of 3.6 ± 1.3 among residents compared to 2.9 ± 1.4 among specialists (*p* = 0.012). No significant differences were found in confidence in the role of AI in management of anesthesia (*p* = 0.099), with an overall score of 3.2 ± 1.4. Resident physicians showed greater confidence in the role of AI in management of surgeries, with an average score of 3.2 ± 1.4 compared to 2.5 ± 1.3 among specialists (*p* = 0.011). The role of AI in event prediction under anesthesia followed the same trend, scoring 3.5 ± 1.4 among resident physicians compared to 2.9 ± 1.4 among specialists (*p* = 0.032), as did the role of AI in decision-making in anesthetic complications and anesthetic crisis, with a score of 3.3 ± 1.4 among residents and a score of 2.8 ± 1.4 among specialists (*p* = 0.034). Furthermore, the utility of AI in patient-centered and equitable pain management yielded a score of 3.5 ± 1.3 among anesthesia residents and a score of 2.9 ± 1.3 among specialists (*p* = 0.016).

Upon investigating concerns about AI. integration in clinical anesthesia, 69 (58.5%) were concerned that the integration of AI. will increase the chance of unethical clinical studies. Moreover, 65 (55.1%) were concerned that the integration of AI will lead to less human–human interaction, while 50 (42.4%) were concerned that integration of AI will lead to more perianesthetic complications. Remarkably, 81 (68.6%) of the included physicians believed that AI-based technology will be more adherent to guidelines in the anesthetic management [Table 2].

## 4. Discussion

The employment of AI in health care depends not only on technological advantages but also on ethical, regulatory, and user attitudes [3]. Success in integrating AI into clinical practice relies on collaboration with anesthesiologists, respecting their expertise, and understanding their opinions about AI. Consequently, understanding anesthesiologists’ attitudes and concerns towards AI utility at tertiary teaching centers is a necessity in order to implement its use in the educational and medical training process, and ultimately applying it in their clinical practice [13,14].

One of the interesting findings is that few interviewees had read a peer-reviewed article on the applications of AI in the field of anesthesia despite a fast-growing body of research literature. Another interesting finding is that more specialists had experience with computer programming and had read an article on the applications of AI. This may be due to the inclusion of young specialists (mean age of included specialists was 39.7 ± 9.5 years) who had recently finished their residency training and started broadening their knowledge and experience. Nonetheless, there was no statistically significant difference between residents and specialists in their confidence in the role of AI in the management of anesthesia.

Anesthesia residents were more inclined to trust in the role of AI in event prediction under anesthesia than consultant anesthesiologists. This is probably due to younger generations being more accepting and trusting of technology than their older counterparts [15,16]. This is a trend that is also clearly visible when it comes to the attitudes of resident physicians versus consultants with regards to role of AI in decision-making in anesthetic complications and anesthetic crisis, and the utility of AI in patient-centered and equitable pain management. Another point worth mentioning is that despite having an average score of 3.2 ± 1.4 for AI’s role in anesthesia management and 3.1 ± 1.4 in decision-making during anesthetic complications, only 29 (24.6%) anesthesiologists rated the role of AI as 5 out of 5. The role of anesthesiologists and their clinical judgment is crucial, and the presence of AI would not replace clinical practitioners in the near future [17].

Even though many of the participants showed inclinations towards the integration of AI into their practice, they did express concerns about possible negative effects it might have. One area of concern is that human–human interaction, a key point in building trust between a doctor and their patient, might be affected by increasing dependency on AI [18]. Another concern of the participants was with the possibility that complication rates may increase with integration of AI: as with any new system, problems may arise at the infancy stages. Those issues may be caused by user error or the inability of a system to detect problems it was not exposed to or designed to handle [19]. A related issue that was not covered by this questionnaire is that if a complication arises from the utilization of AI during anesthesia, the burden of accountability may be challenging to determine, whether it is the anesthesiologist, the hospital, or developer of the AI tool [20]. These uncertainties must be resolved as the integration of AI into anesthesia becomes more widespread. Moreover, governments and bioethics specialists need to establish policies to protect patients’ data and address ethical concerns about the utilization of AI and big data in anesthetic researches [21].

Ultimately, however, the participants in this study thought that AI could more strictly adhere to guidelines in anesthetic management. Likely, previous literature illustrated that AI use is constantly growing and improving, and it has great potential to improve the safety of health care, making provided care more patient-centered, efficient and accessible for patients worldwide [22]. AI can offer major assistance in multiple facets of anesthesia, having a role preoperatively, intraoperatively, or postoperatively. For instance, large data analysis of patient medical records can aid in identifying more specific determinants of patients with possible difficult airways leading to possible difficult intubations [11]. AI can also aid in designing drugs that have better safety profiles and help us further understand how currently available drugs interact with their receptor sites [23]. Postoperatively, AI can be used for better patient pain control [7].

It is worth mentioning that when multiple comparisons are being performed, there is an increased probability that one or more of the significant correlations is a false-positive one, which stands for family-wise error rate (FWER). In the comparison of the role of artificial intelligence (AI) in the perioperative period using Likert scales, 10 comparisons were performed. Therefore, the FWER is 40%. Using Bonferroni adjustment, the adjusted alpha (α) = α/k (number of hypotheses tested) [24], which means the adjusted α must be 0.005. Hence, none of the five-point Likert scales was significant using this new significant threshold for these comparisons. Although we acknowledge the importance of controlling for multiple comparisons to mitigate the risk of type I errors, this approach may be conservative for our exploratory analysis, where our aim was to identify potential areas of interest for further investigations rather than confirmatory hypothesis testing [25]. Moreover, it is equally critical to avoid dismissing potentially valuable insights by overly strict correction methods.

This study had several limitations. Firstly, we aimed to investigate only tertiary teaching hospitals of the Ministry of Health, and hence we excluded military hospitals and private sector hospitals. Therefore, the sample included does not represent all Jordanian anesthesiologists from all sectors. Moreover, the confidence in AI is a dynamic process and is continuously changing, for which follow-up studies must be conducted in the future. Future mixed qualitative–quantitative studies are encouraged, in order to have broader understanding of anesthesiologists’ concerns regarding AI utility.

## 5. Conclusions

AI has the potential to be an effective and revolutionary tool in anesthesia. Success in integrating AI into clinical practice relies on collaborating with anesthesiologists, respecting their knowledge and expertise, understanding their concerns, and improving their confidence in AI integration in the medical field. Hesitancy towards increased dependency on this technology is decreasing with newer generations of practitioners. Hence, this may lead to the wider adoption of AI in anesthesia in the coming years.

## Figures and Tables

**Table 1:** Demographics, knowledge, and experience with AI-based technologies among Jordanian anesthesiologists.

Characteristics		Total (*n* = 118)	Residents (*n* = 74)	Specialists (*n* = 44)	*p* Value
Age (years)		32.7 ± 8.2	28.6 ± 3.0	39.7 ± 9.5	<0.001
Gender	Male	83 (70.3)	48 (64.9)	35 (79.5)	0.091
	Female	35 (29.7)	26 (35.1)	9 (20.5)	
Years of experience as a board-certified anesthesiologist		N.A.	N.A.	7.7 ± 8.8	N.A.
Computer programming experience		69 (58.5)	42 (56.8)	27 (61.4)	0.623
Read any scientific articles about the use of AI in anesthesia		45 (38.1)	24 (32.4)	21 (47.7)	0.098
AI utilization					
None		74 (62.7)	48 (64.9)	26 (59.1)	0.531
AI-based software		37 (31.4)	21 (28.4)	16 (36.4)	0.366
AI-based technology		14 (11.9)	8 (10.8)	6 (13.6)	0.646

AI: artificial intelligence. Numbers are presented as means ± standard deviation or cases (percentage).

**Table 2:** Confidence in AI’s role in different aspects of the perioperative period and concerns about AI integration in clinical anesthesia.

Role of Artificial Intelligence (AI) in the Perioperative Period	Total (*n* = 118)	Residents (*n* = 74)	Specialists (*n* = 44)	*p* Value
Five-point Likert scale to investigate the confidence in AI role in different aspects of the perioperative period				
The role of AI in preoperative evaluation (history taking, physical examination, and investigation)	3.1 ± 1.5	3.2 ± 1.5	2.9 ± 1.5	0.246
The role of AI in preoperative risk stratification	3.8 ± 1.3	3.9 ± 1.3	3.7 ± 1.4	0.547
The role of AI in operating room logistics and management	3.3 ± 1.4	3.6 ± 1.3	2.9 ± 1.4	0.012
The role of AI in management of anesthesia	3.2 ± 1.4	3.3 ± 1.4	2.9 ± 1.5	0.099
The role of AI in management of surgeries	3 ± 1.4	3.2 ± 1.4	2.5 ± 1.3	0.011
The role of AI in events prediction under anesthesia	3.3 ± 1.4	3.5 ± 1.4	2.9 ± 1.4	0.032
The role of AI in decision-making in anesthetic complications and anesthetic crisis	3.1 ± 1.4	3.3 ± 1.4	2.8 ± 1.4	0.034
The role of AI in analysis of anesthetic critical incidents	3.5 ± 1.3	3.6 ± 1.3	3.2 ± 1.3	0.068
The role of AI in patient-centered and equitable pain management	3.3 ± 1.3	3.5 ± 1.3	2.9 ± 1.3	0.016
The role of AI in postoperative follow-up	3.2 ± 1.4	3.4 ± 1.4	2.9 ± 1.3	0.087
Concerns about AI integration in clinical anesthesia				
Concerned that the integration of AI will increase the chance of unethical clinical studies	69 (58.5)	39 (52.7)	30 (68.2)	0.099
Concerned that the integration of AI will lead to less human–human interaction	65 (55.1)	39 (52.7)	26 (59.1)	0.5
Concerned that the integration of AI will lead to more perianesthetic complications	50 (42.4)	29 (39.2)	21 (47.7)	0.364
Believe that AI-based technology will be more adherent to guidelines in the anesthetic management	81 (68.6)	50 (67.6)	31 (70.5)	0.744

Numbers are presented as means ± standard deviation or cases (percentage).

## Data Availability

The data presented in this study are available on request from the corresponding author.

## Supplementary Materials

The following supporting information can be downloaded at https://www.mdpi.com/article/10.3390/jpm14050447/s1. Table S1: Reliability statistics of the questionnaire’s Likert scales.

## References

[1] Mintz Y., Brodie R. (2019). Introduction to artificial intelligence in medicine. Minim. Invasive Ther. Allied Technol..

[2] Botney R. (2008). Improving patient safety in anesthesia: A success story?. Int. J. Radiat. Oncol. Biol. Phys..

[3] Hashimoto D.A., Witkowski E., Gao L., Meireles O., Rosman G. (2020). Artificial Intelligence in Anesthesiology: Current Techniques, Clinical Applications, and Limitations. Anesthesiology.

[4] Kühl N., Schemmer M., Goutier M., Satzger G. (2022). Artificial intelligence and machine learning. Electron. Mark..

[5] Glen J.B. (1998). The development of ‘Diprifusor’: A TCI system for propofol. Anaesthesia.

[6] Ngai L., Thierry C., Sophie H., Alain L., Nathalie B., Corinne D., Bernard T., Laurent B., Emmanuel S., Sessler D.I. (2011). Closed-loop coadministration of propofol and remifentanil guided by bispectral index: A randomized multicenter study. Anesth. Analg..

[7] Singhal M., Gupta L., Hirani K. (2023). A Comprehensive Analysis and Review of Artificial Intelligence in Anaesthesia. Cureus.

[8] Wijnberge M., Geerts B.F., Hol L., Lemmers N., Mulder M.P., Berge P., Schenk J., Terwindt L.E., Hollmann M.W., Vlaar A.P. (2020). Effect of a Machine Learning–Derived Early Warning System for Intraoperative Hypotension vs. Standard Care on Depth and Duration of Intraoperative Hypotension During Elective Noncardiac Surgery: The HYPE Randomized Clinical Trial. JAMA.

[9] Singh M., Nath G. (2022). Artificial intelligence and anesthesia: A narrative review. Saudi J. Anaesth..

[10] Antel R., Sahlas E., Gore G., Ingelmo P. (2023). Use of artificial intelligence in paediatric anaesthesia: A systematic review. BJA Open.

[11] Song B., Zhou M., Zhu J. (2023). Necessity and Importance of Developing AI in Anesthesia from the Perspective of Clinical Safety and Information Security. Med. Sci. Monit..

[12] Benjamens S., Dhunnoo P., Meskó B. (2020). The state of artificial intelligence-based FDA-approved medical devices and algorithms: An online database. NPJ Digit. Med..

[13] Mir M.M., Mir G.M., Raina N.T., Mir S.M., Mir S.M., Miskeen E., Alharthi M.H., Alamri M.M.S. (2023). Application of Artificial Intelligence in Medical Education: Current Scenario and Future Perspectives. J. Adv. Med. Educ. Prof..

[14] Grunhut J., Marques O., Wyatt A.T.M. (2022). Needs, challenges, and applications of artificial intelligence in medical education curriculum. JMIR Med. Educ..

[15] Olson K.E., O’Brien M.A., Rogers W.A., Charness N. (2011). Diffusion of Technology: Frequency of Use for Younger and Older Adults. Ageing Int..

[16] Nawaz I.Y. (2020). Characteristics of millennials and technology adoption in the digital age. Handbook of Research on Innovations in Technology and Marketing for the Connected Consumer.

[17] Pham F.M. (2023). Artificial intelligence-supported systems in anesthesiology and its standpoint to date—A review. Open J. Anesthesiol..

[18] Wienrich C., Latoschik M.E. (2021). Extended artificial intelligence: New prospects of human-ai interaction research. Front. Virtual Real..

[19] Khan B., Fatima H., Qureshi A., Kumar S., Hanan A., Hussain J., Abdullah S. (2023). Drawbacks of Artificial Intelligence and Their Potential Solutions in the Healthcare Sector. Biomed. Mater. Devices.

[20] Kavian J.A., Wilkey H.L., Patel P.A., Boyd C.J. (2023). Harvesting the Power of Artificial Intelligence for Surgery: Uses, Implications, and Ethical Considerations. Am. Surg..

[21] Anom B.Y. (2020). Ethics of Big Data and artificial intelligence in medicine. Ethics Med. Public Health.

[22] Rajpurkar P., Chen E., Banerjee O., Topol E.J. (2022). AI in health and medicine. Nat. Med..

[23] Blanco-González A., Cabezón A., Seco-González A., Conde-Torres D., Antelo-Riveiro P., Piñeiro Á., Garcia-Fandino R. (2023). The Role of AI in Drug Discovery: Challenges, Opportunities, and Strategies. Pharmaceuticals.

[24] Lee S., Lee D.K. (2018). What is the proper way to apply the multiple comparison test?. Korean J. Anesthesiol..

[25] Armstrong R.A. (2014). When to use the Bonferroni correction. Ophthalmic Physiol. Opt..

