# Supplementary material for: Attitudes of Jordanian Anesthesiologists and Anesthesia Residents towards Artificial Intelligence: A Cross-Sectional Study
Source: J Pers Med. 2024 Apr 25;14(5):447. doi: 10.3390/jpm14050447 (PMC11121815; doi:10.3390/jpm14050447)
Supplement: Supplementary file 1 [file jpm-14-00447-s001.zip › jpm-2921266-supplementary.pdf]

Table S1. Reliability statistics of the questionnaire’s Likert scales.

Item	Scale Mean if Item Deleted	Scale Variance if Item Deleted	Corrected Item-Total Correlation	Squared Multiple Correlation	Cronbach's Alpha if Item Deleted
1- The role of A.I. in preoperative evaluation (history taking, physical examination, and investigation)	29.6	104.0	0.797	0.765	0.936
2- The role of A.I. in preoperative risk stratification	29.0	110.1	0.656	0.535	0.942
3- The role of A.I. in operating room logistics and management	29.4	107.0	0.754	0.601	0.938
4- The role of A.I. in management of anesthesia	29.6	103.5	0.842	0.753	0.933
5- The role of A.I. in management of surgeries	29.8	106.2	0.760	0.641	0.937
6- The role of A.I. in events prediction under anesthesia	29.5	104.4	0.817	0.724	0.935
7- The role of A.I. in decision making in anesthetic complications and anesthetic crisis	29.7	105.1	0.800	0.764	0.935
8- The role of A.I. in analysis of anesthetic critical incidents	29.3	109.3	0.697	0.632	0.940
9- The role of A.I. in patient centered and equitable pain management	29.5	106.0	0.821	0.707	0.935
10- The role of A.I. in postoperative follow-up	29.6	107.9	0.701	0.585	0.940
